# Testing Toughened Glass

**Published:** 1875-09

**Authors:** 


TESTING TOUGHENED GLASS.—Glass is subject to sudden, sharp blows, either from articles falling down on other substances or from extraneous bodies falling upon or being brought in contact with them. Hence it is clear that to obtain a true estimate of the new process, glass must be subjected to tests which fairly represent the conditions of the accidents to which it is ordinarily exposed. This estimate has been arrived at repeatedly by placing pieces of plate-glass in a frame and allowing weights to fall on them from given heights. One experiment from a number—and which was made publicly—will illustrate this test: A piece of ordinary glass, six inches long by five inches wide, and one-fourth of an inch thick, was placed in a small frame which supported the glass around its edges, and kept its under side about half an inch from the floor. A four-ounce weight was dropped on it from a height of one foot, and the glass was broken. A piece of toughened glass of corresponding dimensions was then placed in the frame and the same weight dropped upon it several times from a height of ten feet, but without fracturing the glass. An eight-ounce weight was then substituted, and repeatedly dropped upon the glass from the same height as before, and with the same result, no impression whatever being made upon it. The eight-ounce weight was then thrown violently upon it several times, but without damaging it. Its destruction, however, was finally accomplished by means of a hammer. Perhaps the most crucial test to which toughened glass could be put would be to let it fall on iron. This has been done, and in public too. A thin glass plate was dropped from a height of four feet on to an iron grating, from which it rebounded about one foot, sustaining no injury whatever.—*Popular Science Monthly for September.*

